# Supplementary material for: Implementation of a volunteer contact tracing program for COVID-19 in the United States: A qualitative focus group study
Source: PLoS One. 2021 May 5;16(5):e0251033. doi: 10.1371/journal.pone.0251033 (PMC8099418; doi:10.1371/journal.pone.0251033)
Supplement: S1 Text — (DOCX) [file pone.0251033.s002.docx]

S1 Text. Semi-Structured Focus Group Guide: Contact Tracing Volunteers

Intro

*[Obtain verbal consent.]*

**Thank you for agreeing to participate in this study. Your experience working on the NHHD contact tracing program makes you an expert on this topic, and we would like to learn from you. This information will help the health department improve their contact tracing program and may also help other health departments by giving them advice about best ways to do contact tracing for COVID-19.**

Domains

- Working in a volunteer program
  - **What made you decide to volunteer with this program?** (peer pressure, personal motivation)
  - **Tell me a little about what’s it been like to work as a volunteer in this program.** (self-efficacy)
  - **What was it like for you in balancing this job with your other responsibilities?**
  - **How do you think this program in general, and your role in particular, evolved over time?**
  - **Recommendations?**
- Successes/Challenges
  - **What do you think went well in the calls you made?**
  - **What did you have difficulty with in those calls?**
- Training and Unexpected Experiences
  - **What is your opinion of the training you received?**
  - **What did you think of the Training Format and Content?**
  - **What were some of the unexpected experiences you encountered while doing the contact tracing/while volunteering with this program?**
  - **What tools or training do you wish you would have had?**
  - **What infrastructure or support do you wish the program had offered?**
  - **Recommendations?**
- Hopes and Concerns regarding the future
  - **How do you foresee the program changing over time?**
  - **How do you foresee your involvement in the program changing over time?**
  - **Recommendations?**
- **Is there anything else that you would like to add that we haven’t talked about, but you think would be important for me to know?**

**Thank you for your time!**
